# Adrenaline aggravates lung injury caused by liver ischemia–reperfusion and high-tidal-volume ventilation in rats

**DOI:** 10.1186/s40560-016-0130-y

**Published:** 2016-01-22

**Authors:** Shuhei Ota, Takuya Yazawa, Kentaro Tojo, Yasuko Baba, Munehito Uchiyama, Takahisa Goto, Kiyoyasu Kurahashi

**Affiliations:** Department of Anesthesiology and Critical Care Medicine, Yokohama City University Graduate School of Medicine, 3-9 Fukuura, Kanazawa-ku, Yokohama, Kanagawa 236-0004 Japan; Department of Pathology, Yokohama City University Graduate School of Medicine, 3-9 Fukuura, Kanazawa-ku, Yokohama, Kanagawa 236-0004 Japan

**Keywords:** Acute respiratory distress syndrome, Ventilator-induced lung injury, Adrenaline cytokines, Liver ischemia–reperfusion

## Abstract

**Background:**

We often administer adrenaline to improve hypotension of patients undergoing systemic inflammation that is not treated with volume resuscitation. The effects of adrenaline on injured lungs during shock status have not been elucidated. We previously demonstrated that hepatic ischemia–reperfusion followed by high-tidal-volume ventilation-induced systemic inflammation, hypotension, and lung injury in rats. Using this animal model, we investigated the effects of adrenaline on lung injury and hemodynamics.

**Methods:**

Anesthetized rats were ventilated and underwent hepatic inflow interruption for 15 min twice. After the second liver ischemia–reperfusion, the tidal volume was increased to 24 ml · kg^−1^ body weight from 6 ml · kg^−1^, and 12 rats in each group were observed for 360 min after reperfusion with or without continuous intravenous adrenaline administration. Extra fluid was administered according to the decline in the arterial blood pressure.

**Results:**

Adrenaline administration significantly reduced the volume of intravenous resuscitation fluid. The wet-to-dry weight ratio of the lungs was higher (7.53 ± 0.37 vs. 4.63 ± 0.35, *P* < 0.001), the partial oxygen pressure in arterial blood was lower (213 ± 48 vs. 411 ± 33, *P* = 0.004), and the tumor necrosis factor-α concentration in bronchoalveolar lavage (BAL) fluid was higher (10^2.64^ ± 10^0.22^ vs. 10^1.91^ ± 10^0.27^, *P* = 0.015), with adrenaline. Histopathological examinations revealed marked exudation in the alveolar spaces in rats receiving adrenaline.

**Conclusions:**

Continuous administration of adrenaline partially prevented a rapid decline in blood pressure but deteriorated lung injury in a rat model of liver ischemia–reperfusion with high-tidal-volume ventilation. A possibility that adrenaline administration aggravate ventilator-induced lung injury during systemic inflammation should be considered.

## Background

Systemic inflammation is one of the main causes of adverse events after a major surgery. The lungs are one of the most susceptible organs to systemic inflammation during and after a major surgery, including liver transplant [1], cardiac surgery [2], spine surgery [3], and thoracic surgery [4]. Pulmonary complications after a major surgery include pleural effusion, atelectasis, pulmonary edema, respiratory distress syndrome, and pneumonia [1].

Positive inotropes and vasopressors such as adrenaline and volume resuscitation are often used to prevent blood pressure decline caused by systemic inflammation during and after a major surgery. Previous studies demonstrated that β-adrenergic agonists increase alveolar liquid clearance (ALC) [5, 6]. On the other hand, systemic administration of β-adrenergic agonist increases cardiac output and aggravates pulmonary edema on oleic acid-induced lung injury [7]. Furthermore, drugs that have α-adrenergic property may impair lung edema due to increased pulmonary vascular resistance [8]. From these observations, administration of adrenaline, a potent α- and β-adrenergic agonist, for systemic inflammation and shock status may affect lung injury; however, the effect of continuous administration of adrenaline on injured lungs during shock status have not yet been elucidated.

We had shown that repeated hepatic inflow obstruction and reperfusion concomitant with large tidal volume (V_T_) ventilation causes shock and mild lung injury in rats [9]. Using this animal model, we investigated the effect of adrenaline on hemodynamics and lung injury.

## Methods

### Animal preparation

All protocols for animal experiments were approved by the Animal Research Committee of the Yokohama City University, Yokohama, Japan (approval numbers: 03–65 and 04–68). Specific pathogen-free male Sprague–Dawley rats, weighing 320 to 390 g (aged 9 to 11 weeks) (Japan SLC, Shizuoka, Japan), were used for all experiments. We used the same general protocol as in our previous study [9]. In brief, rats were mechanically ventilated through a tracheotomy under pentobarbital anesthesia. The tidal volume was set to 6 ml · kg^−1^ from the beginning of ventilation to the end of the second hepatic inflow obstruction. Ventilation frequency was adjusted to maintain the arterial carbon dioxide tension between 35 and 50 Torr. Hydroxyethylated starch 70000 (Fresenius Kabi Japan, Tokyo, Japan) was infused at a rate of 10 ml · kg^−1^ · h^−1^ for the first hour and then at a rate of 5 ml · kg^−1^ · h^−1^. Arterial blood was withdrawn at 30, 60, and 120 min after reperfusion and every 2 h thereafter for a blood gas analysis and measurement of plasma tumor necrosis factor-α (TNF-α) levels. The volume of withdrawn blood was replaced by the same volume of Ringer’s lactate solution into the artery. In addition, 1 ml of the solution was administered intra-arterially at an interval of 10, 5, 2, or 1 min when systolic arterial blood pressure (sABP) decreased to 100, 90, 80, or 70 mmHg, respectively. Six hours after reperfusion, rats were deeply anesthetized and euthanized.

### Liver ischemia–reperfusion

Hepatic inflow was interrupted by placing a vascular clip on the hepatic artery and portal vein. Ischemia was induced twice for 15 min with an interval of 5 min between the two hepatic blood flow interruptions.

### Experimental groups

A total of 24 rats were used to evaluate lung injury and systemic responses to liver ischemia–reperfusion. After the second liver ischemia–reperfusion, the tidal volume was set to 24 ml · kg^−1^ and this time point was defined as time 0. Rats were assigned to a group receiving 0.5 ml · h^−1^ of saline (*n* = 12) or a group receiving adrenaline (*n* = 12) according to the protocol described below. We shared the data of rats in the saline group with the previous study [9] to reduce the number of newly sacrificed animals because the protocol was set to be the same. The experiments were conducted from June 2003 to January 2004 in the saline group and from September 2004 to November 2004 in the adrenaline group.

### Adrenaline administration protocol

Adrenaline was administered initially at a rate of 0.05 μg · kg^−1^ · min^−1^ when sABP became less than 120 mmHg and was increased by 0.05 μg · kg^−1^ · min^−1^ after 30 min of the lock-up time when sABP became less than 120, 110, 110, 100, 90, 80, or 70 mmHg on or after time 150, 180, 210, 240, 270, 300, and 330 min, respectively. No further increment in the infusion of adrenaline was made when the infusion rate reached 0.3 μg · kg^−1^ · min^−1^ because the dose of adrenaline is around 0.2 μg · kg^−1^ · min^−1^ for clinical use in humans [10].

### Measurement of lung injury

Lung injury was assessed in two different ways. The right lungs of one subset of rats (*n* = 9) from each group were used to determine the wet-to-dry weight ratio of the lungs, which indicates lung edema. The ratio was calculated according to a previously established method [9, 11, 12]. The right lungs of another subset of rats (*n* = 3) from each group were used for a histopathological examination. The formalin-fixed and paraffin-embedded sections were processed for hematoxylin and eosin staining. The pathologist who was blinded to the experimental groups assessed and scored the degree of perivascular and peribronchial edema, number of leukocytes in the pulmonary capillaries, nuclear swelling in the epithelial cells, and cellular infiltration and exudation into the alveoli.

### Bronchoalveolar lavage

Left lungs of eight rats in each group underwent bronchoalveolar lavage (BAL) fluid collection 6 h after reperfusion or at the time rats died. BAL fluid was collected twice with 1.5 ml of phosphate-buffered saline (PBS) containing 0.1 % EDTA per lavage. The fluid was then centrifuged at 3400*g* at 4 °C for 20 min, and the supernatant was stored at −80 °C until use.

### Assay for TNF-α in BAL fluid and blood

A biological TNF-α assay was performed using the mouse sarcoma cells, WEHI-13VAR (CRL2148; American Type Cell Culture, Manassas, VA), as previously reported [9, 13]. The TNF-α activity of each sample was calculated by comparing absorbance to that of a standard curve made from dilutions of rat TNF-α (PharMingen, San Diego, CA) between 1.2 pg · ml^−1^ and 1250 pg · ml^−1^. The lower limit of detection of this assay was 1.2 pg · ml^−1^. The assays were performed between September 2004 and December 2004.

### Statistical analysis

All data were analyzed using the Statcel 2nd edition (OMS Publishing, Tokorozawa, Japan). Data obtained at a single time point during the experiment were presented as mean ± standard error of the mean, and significance was assessed using the Student’s *t* test. The data obtained repeatedly were analyzed using the two-way repeated measure of analysis of variance followed by Student’s *t* test with Bonferroni correction. Because some rats died between 240 and 360 min, the analysis was performed for the time period of up to 240 min. A *P* value of less than 0.05 was considered significant.

## Results

### Survival and observational period

Eight rats in the saline group and three in the adrenaline group died between 300 and 360 min after reperfusion. Stated in detail, 300, 300, 313, 321, 330, 337, 342, and 349 are the dead points for rats in the saline group and 300, 337, and 359 are those for rats in the adrenaline group.

### Hemodynamics changes

**Fig. 1:**
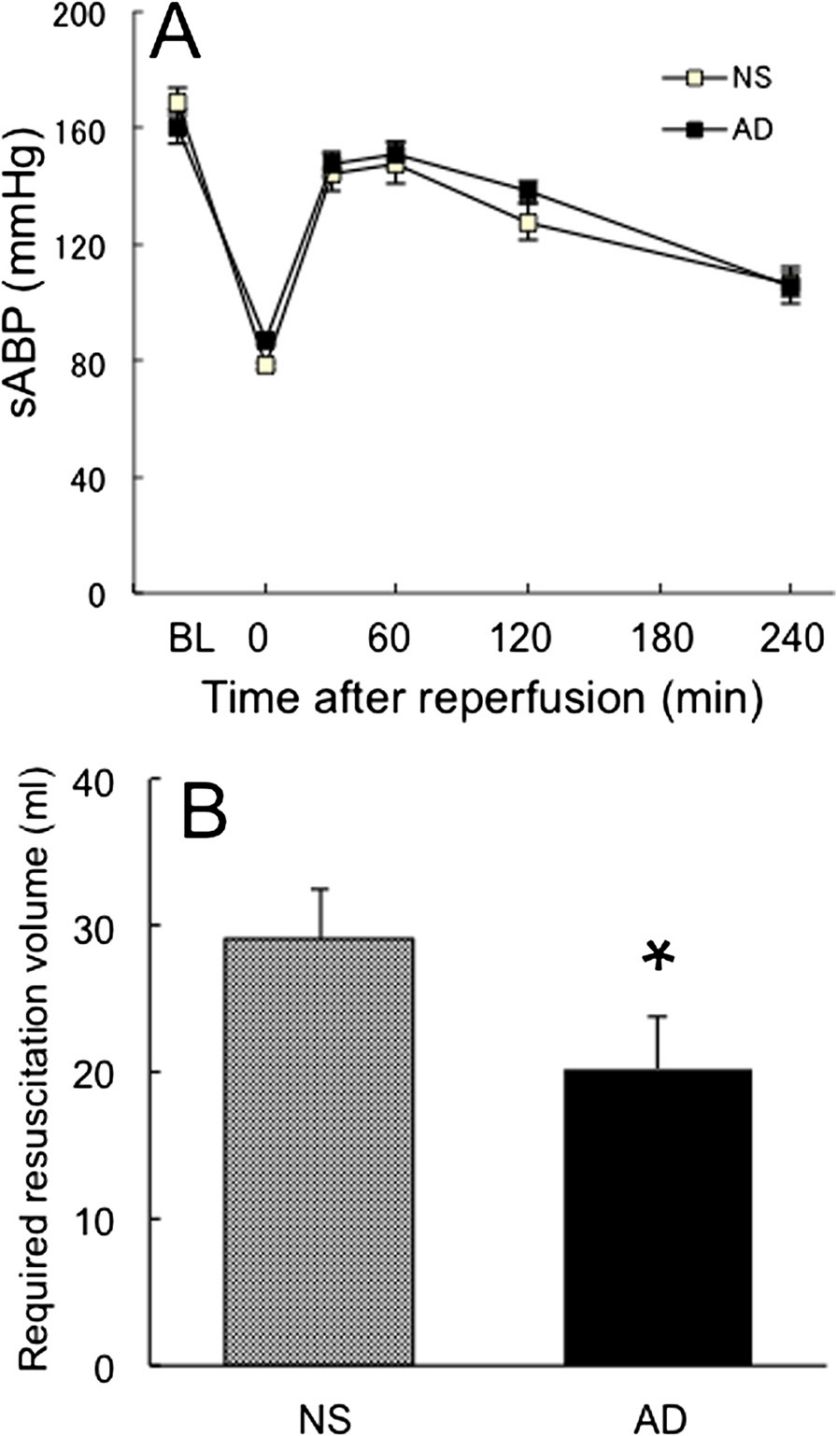
Systolic arterial blood pressure (sABP) and required resuscitation volume. **a** sABP as an indicator of hemodynamics (reproduced with modification with the permission of the American Physiological Society [9]). **b** Total volume of additional bolus injection of the Ringer’s lactate solution to maintain blood pressure. BL: baseline value taken just before the interruption of hepatic inflow. Reperfusion was started at time 0. Mean ± standard error of the mean. NS: hepatic ischemia–reperfusion and saline infusion. AD: hepatic ischemia–reperfusion and adrenaline infusion. *P < 0.05 vs. NS group

Hepatic inflow interruption caused a rapid and progressive decrease in sABP. It returned to the baseline values just after reperfusion but then gradually decreased during the 6-h reperfusion period. sABP values at 240 min in both groups were significantly lower than those at baseline. There was no difference in sABP between the saline and adrenaline groups (Fig. 1a). Extra fluid was given to rats to maintain ABP during liver ischemia and reperfusion periods, and significantly less volume was infused in the adrenaline group than in the saline group (Fig. 1b).

### Oxygenation

**Fig. 2:**
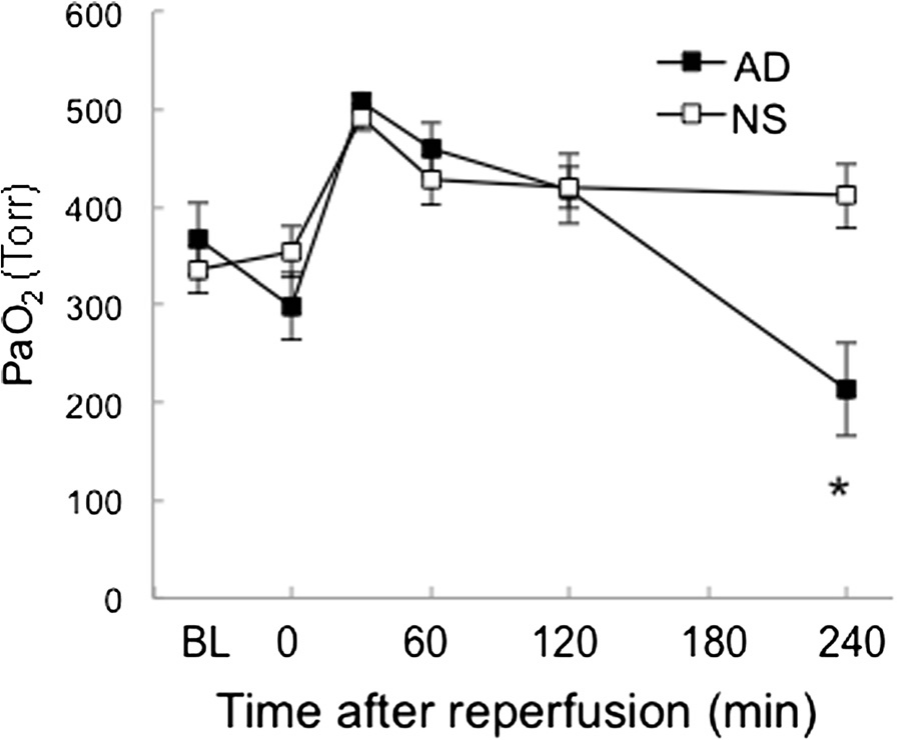
Changes in arterial partial pressure of oxygen (PaO_2_). PaO_2_, an indicator of oxygenation, was significantly lower in the adrenaline group compared with the saline group at 240 min. BL: baseline value taken just prior to the interruption of hepatic inflow. Reperfusion was started at time 0. Mean ± standard error of the mean. NS: hepatic ischemia–reperfusion and saline infusion. AD: hepatic ischemia–reperfusion and adrenaline infusion. *P < 0.05 vs. NS group

Oxygenation deteriorated in both groups (Fig. 2). Arterial partial pressure of oxygen (PaO_2_) was significantly lower in the adrenaline group than in the saline group at 240 min.

### Wet-to-dry weight ratio of the lungs

**Fig. 3:**
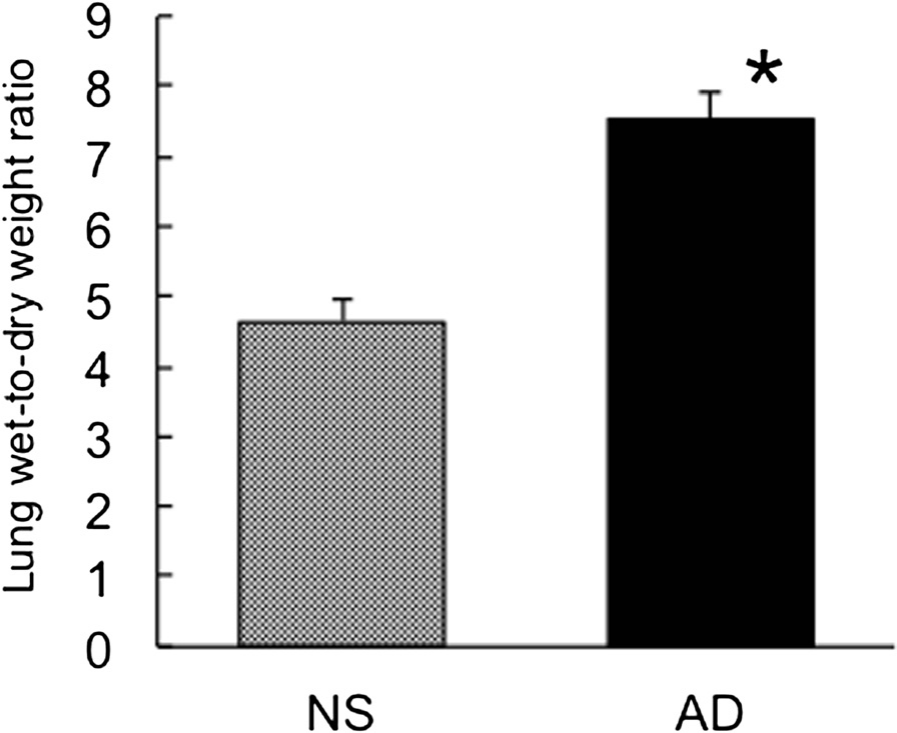
Assessment of lung edema. Wet-to-dry weight ratio of the lungs was assessed as an indicator of lung edema. Mean ± standard error of the mean. NS: hepatic ischemia–reperfusion and saline infusion. AD: hepatic ischemia–reperfusion and adrenaline infusion. *P < 0.05 vs. NS group (reproduced with modification with permission of the American Physiological Society [9])

Lungs were harvested at 360 min or when rats died, 300 to 359 min after the reperfusion. The wet-to-dry weight ratio of the lungs was significantly higher in the adrenaline group compared with the saline group (Fig. 3).

### Histopathological study

**Fig. 4:**
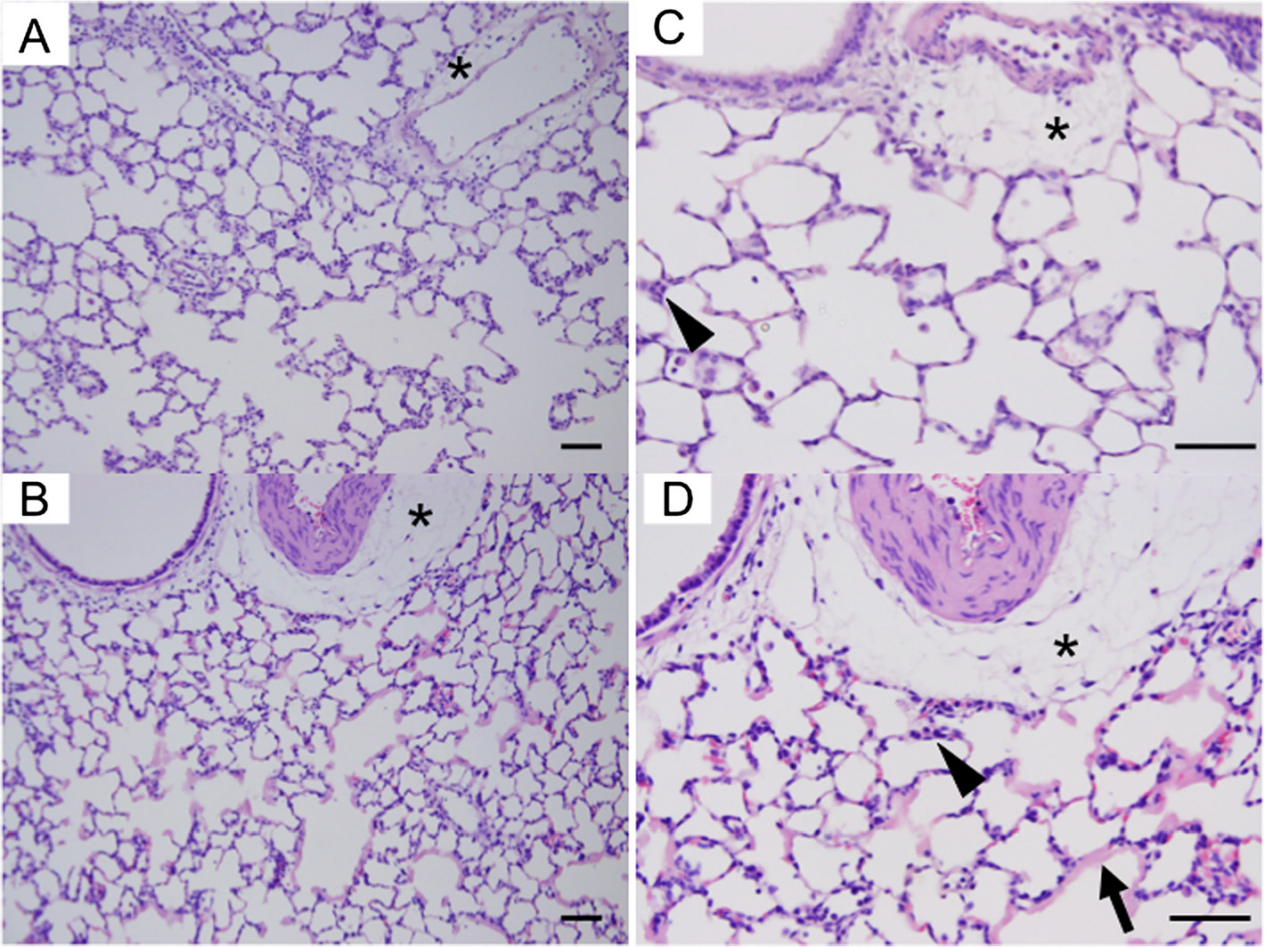
Representative micrographs of the lung tissue stained with hematoxylin and eosin. **a**, **c** lung of hepatic ischemia–reperfusion and saline infusion group. Marked perivascular space thickening (*) is seen. **b**, **d** lung of the ischemia–reperfusion and adrenaline infusion group. A small vessel obstruction due to the aggregation of leukocytes and microthrombi (*arrowhead*) and marked exudation in the alveolar spaces (*arrow*) are seen in addition to perivascular space thickening (*). *Black bars*: 50 μm

The lungs harvested at the same time as the wet-to-dry weight ratio measurements were histopathologically examined using hematoxylin and eosin stained sections (Fig. 4). Perivascular edema was pronounced in both groups. Adhesion of leukocytes to the vascular walls and the obstruction of small vessels with leukocytes and microthrombi were also detected in both groups. Marked exudation was found in the alveolar spaces in the adrenaline group with the degeneration and desquamation of pneumocytes. These exudative changes were not observed in the saline group. These pathological findings are scored and presented in Table 1.

**Table 1:** Histopathological evaluation

	Edema	Capillary	Epithelium	Infiltrates	Exudation
	1	1	1	0	0
NS (*n* = 3)	2	2	1	0	0
	2	2	1	0	0
	2	2	2	0	2
AD (*n* = 3)	2	2	2	0	2
	2	2	2	1	2

Perivascular and peribronchial edema (Edema), leukocytes in pulmonary capillary (Capillary), nuclear swelling in epithelial cell (Epithelium), cellular infiltration into alveoli (Infiltrates), and exudation into alveoli (Exudation) were assessed by a pathologist in a blinded manner. These observations were scored using three grades: 0, no or mild change; 1, moderate change; and 2, marked change

### TNF-α concentrations in plasma and BAL fluid

**Fig. 5:**
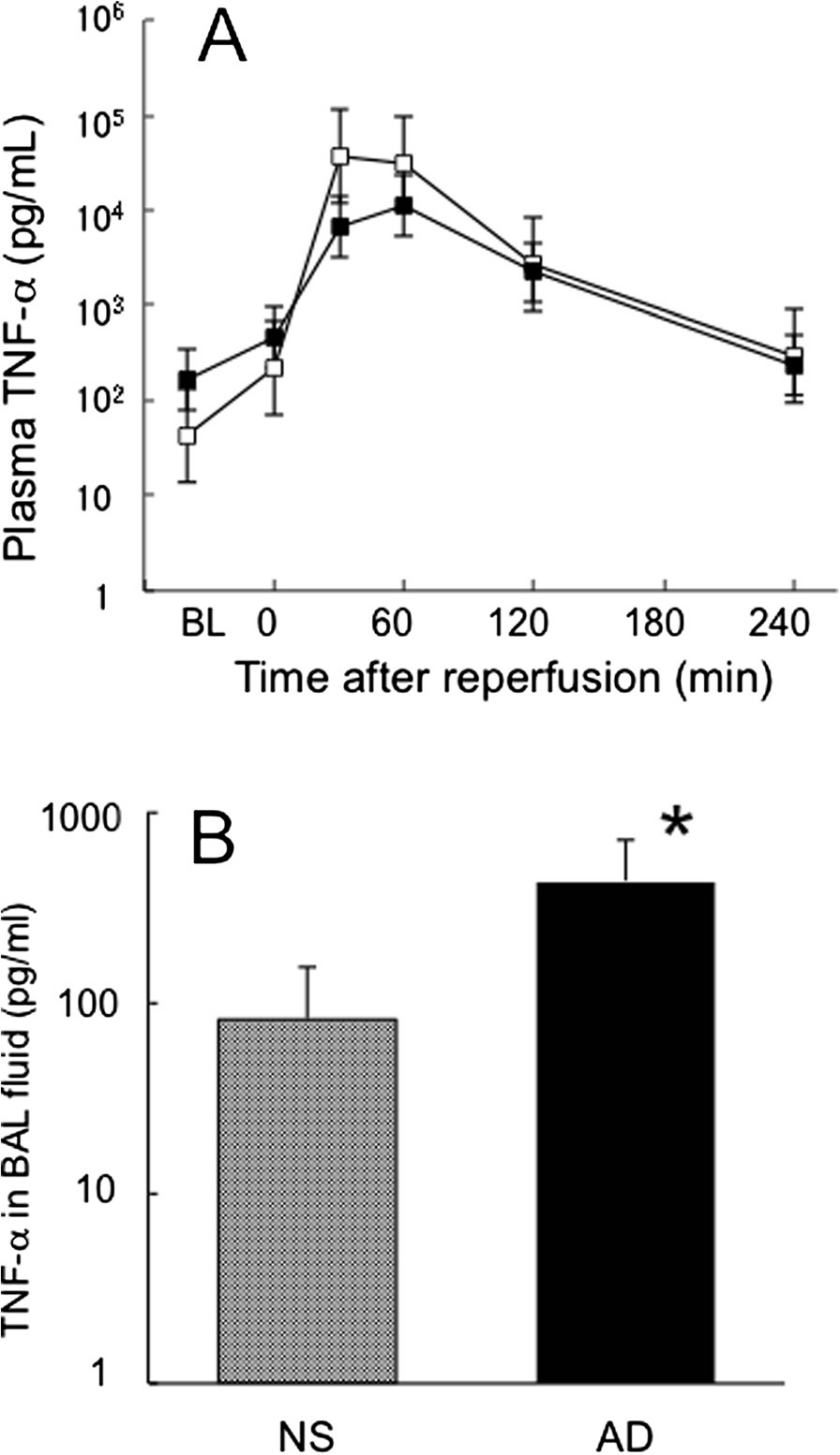
Tumor necrosis factor-α (TNF-α) concentrations in plasma and BAL fluid. **a** Plasma TNF-α concentration increased at early time point after reperfusion and declined thereafter. There was no difference in the plasma TNF-α concentration between the groups. **b** Concentration of tumor necrosis factor-α (TNF-α) in bronchoalveolar lavage (BAL) fluid. BAL was performed 6 h after reperfusion or at the time rats died, between 300 to 359 min after reperfusion. TNF-α in BAL fluid was significantly higher in the adrenaline group than in the saline group. Mean ± standard error of the mean. NS: hepatic ischemia–reperfusion and saline infusion. AD: hepatic ischemia–reperfusion and adrenaline infusion. *P < 0.05 vs. NS group (reproduced with modification with permission of the American Physiological Society [9])

Plasma TNF-α concentrations transiently increased between 30 and 60 min after reperfusion and declined thereafter in both groups (Fig. 5a). There was no difference in the TNF-α concentration between the two groups at any time points. The TNF-α concentration in BAL fluid was significantly higher in the adrenaline group compared with the saline group (Fig. 5b).

## Discussion

The administration of adrenaline to rats that underwent shock after hepatic inflow obstruction and reperfusion under high-tidal-volume ventilation caused pulmonary edema and deteriorated oxygenation. Considering the fact that administration of adrenaline reduced the volume of resuscitation fluid during the reperfusion period, adrenaline administration but not fluid overload is the cause for pulmonary edema.

Previous studies demonstrated that β-adrenergic receptor agonists increase ALC in normal lungs in rats [5, 14] and dogs [15]. In a pathological model, Saldías et al. [16] showed that terbutaline and isoproterenol increased lung edema clearance in ventilator-associated lung injury induced by high-tidal-volume ventilation in rats. Using human lung sections resected during surgery for lung cancer, Sakuma et al. demonstrated β-adrenergic stimulation increased the rate of alveolar liquid clearance [17]. Sartori et al. [18] demonstrated the clinical benefit of prophylactic inhalation of β-adrenergic agonists for reducing high-altitude pulmonary edema in humans. However, in a recent randomized placebo-controlled trial, the effects of the β_2_-adrenergic receptor agonist, albuterol, in acute lung injury did not increase ventilator-free days or reduce the rate of death before hospital discharge [19]. Mutlu et al. [20] reviewed papers and discussed that β-adrenergic receptor function may be impaired in pathological lungs. This concept partly explains the inability of adrenaline to decrease lung edema in the present study. In addition to β-adrenergic property, adrenaline has α-adrenergic property. It is well known that adrenaline increases pulmonary vascular resistance and decreases pulmonary vascular compliance through α-adrenergic receptor-mediated stimulation [8]. Rassler et al. demonstrated in rat experiments that α-adrenergic agonists induced pulmonary edema by increasing total peripheral vascular resistance and right ventricular systolic pressure [21]. Krishnamoorthy et al. [22] demonstrated that a bolus injection of adrenaline to intact rats impaired pulmonary oxygen exchange, which was blunted by an α-adrenergic receptor antagonist. These observations suggest that adrenaline have the potential to promote pulmonary edema through α-adrenergic receptor stimulation.

TNF-α concentration in plasma increased similarly in both groups; however, that in BAL fluid was about 10 times higher in the adrenaline group than in the saline group. Briot et al. [7] demonstrated that the leakage of macromolecules through the lung epithelial barrier increased when cardiac output increased with terbutaline infusion in oleic acid-induced lung injury. Our result is consistent with the previous studies in terms that adrenaline enhances translocation of macromolecules including TNF-α protein through the alveolar-capillary barrier during lung injury. On the other hand, there is a possibility that TNF-α production increased in the lungs in the AD group, reason for which is unclear and further investigation is warranted.

In clinical practice, adrenaline is often administered during severe hypotension; therefore, it is important to demonstrate the effects of adrenaline on lung physiology during shock status. The present study demonstrated potential harmful effects of adrenaline on lung injury during shock status. Further studies are needed to elucidate the underlying mechanisms of the effect of adrenaline and other inotropes or vasopressors on lung injury. To our knowledge, this is the first study that has documented the effects of adrenaline on lung injury during surgery.

There are some limitations to this study. Although it is now standard to limit the tidal volume during mechanical ventilation in patients with ARDS, we ventilated rats with high-tidal volume. Because the aim of the present study was to investigate the effects of adrenaline under conditions of unstable hemodynamics, we used the protocol by which experimental animals experience hypotension and lung injury. The present study may not be a suitable alternative to a clinical model for ventilator strategy for lung injury. Second, some rats (more in the saline group) died before the completion of the 6-h experiment, suggesting that there are biases in data obtained from the lungs harvested at the end of the experiment. However, most rats that did not survive till the end of the experiment died close to 360 min suggesting that the effects of data obtained from rats died before the completion of the study is minimal if present.

## Conclusions

In conclusion, an intravenous continuous administration of adrenaline after hepatic inflow obstruction significantly deteriorated pulmonary edema, impaired oxygenation, and increased the alveolar TNF-α concentration during high-tidal volume ventilation. A possibility that adrenaline administration might aggravate ventilator-induced lung injury during systemic inflammation should be considered.
